# Effusanin E Suppresses Nasopharyngeal Carcinoma Cell Growth by Inhibiting NF-κB and COX-2 Signaling

**DOI:** 10.1371/journal.pone.0109951

**Published:** 2014-10-21

**Authors:** Mingzhu Zhuang, Mouming Zhao, Huijuan Qiu, Dingbo Shi, Jingshu Wang, Yun Tian, Lianzhu Lin, Wuguo Deng

**Affiliations:** 1 College of Light Industry and Food Sciences, South China University of Technology, Guangzhou, China; 2 State Key Laboratory of Oncology in South China, Sun Yat-Sen University Cancer Center, Guangzhou, China; 3 State Key Laboratory of Pulp and Paper Engineering, South China University of Technology, Guangzhou, China; University of Hawaii Cancer Center, United States of America

## Abstract

*Rabdosia serra* is well known for its antibacterial, anti-inflammatory and antitumor activities, but no information has been available for the active compounds derived from this plant in inhibiting human nasopharyngeal carcinoma (NPC) cell growth. In this study, we isolated and purified a natural diterpenoid from *Rabdosia serra* and identified its chemical structure as effusanin E and elucidated its underlying mechanism of action in inhibiting NPC cell growth. Effusanin E significantly inhibited cell proliferation and induced apoptosis in NPC cells. Effusanin E also induced the cleavage of PARP, caspase-3 and -9 proteins and inhibited the nuclear translocation of p65 NF-κB proteins. Moreover, effusanin E abrogated the binding of NF-κB to the COX-2 promoter, thereby inhibiting the expression and promoter activity of COX-2. Pretreatment with a COX-2 or NF-κB-selective inhibitor (celecoxib or ammonium pyrrolidinedithiocarbamate) had an additive effect on the effusanin E-mediated inhibition of proliferation, while pretreatment with an activator of NF-κB/COX-2 (lipopolysaccharides) abrogated the effusanin E-mediated inhibition of proliferation. Effusanin E also significantly suppressed tumor growth in a xenograft mouse model without obvious toxicity, furthermore, the expression of p50 NF-κB and COX-2 were down-regulated in the tumors of nude mice. These data suggest that effusanin E suppresses p50/p65 proteins to down-regulate COX-2 expression, thereby inhibiting NPC cell growth. Our findings provide new insights into exploring effusanin E as a potential therapeutic compound for the treatment of human nasopharyngeal carcinoma.

## Introduction

Nasopharyngeal carcinoma (NPC) is a relatively uncommon, malignant, head and neck cancer that is found worldwide but is highly prevalent in South China and Southeast Asia [1]. It frequently occurs in the Guangdong area, China, where the annual incidence reaches 25 cases per 100,000 [2]. A combination of radiotherapy and adjuvant chemotherapy is now the standard treatment for NPC. However, the 5-year survival rate is only 50–60% due to the frequency of distant metastasis and local recurrence and the long-term secondary effects of radiotherapy and chemotherapy [3]. In addition, these methods may sometimes cause severe acute toxicity and even increased incidence of late complications without obvious survival benefits [4]. Currently, the use of natural, synthetic or biologic chemicals has been considered as effective cancer chemopreventions in the prevention, suppression or delay of the carcinogenesis process [5].

The plant *Rabdosia serra* (*R. serra*), which is called Xihuangcao in Chinese, has been used in traditional Chinese medicine for centuries as a characteristic plant resource in south China. The genus *R. serra* is well known for its antibacterial, antiviral, anti-inflammatory and antitumor activities [6], is a rich source of diterpenoids and is widely distributed in China. It has been shown that some chemicals isolated from this plant have inhibitory effects on cancer cell growth in vitro and tumor growth in vivo. The strong cytotoxicities of diterpenoids isolated from *R. serra*, such as nodosin, oridonin and lasiokaurin, against human cancer cells were verified in previous investigations. Diterpenoid could effectively reduce the accumulation of COX-2 protein to exhibit cytotoxicity against cancer cells [7]. It also has been shown that diterpenoids (quercetin and oridonin) derived from I. Japonicus exerted its anticancer properties by regulating the NF-κB signaling pathways [8]-[10]. Additionally, it is known that effusanin C inhibits inflammatory responses via blocking NF-κB signaling in monocytes [11].

COX-2 is an inducible enzyme that plays a critical role in multiple pathophysiological processes such as inflammation, atherosclerosis, tissue injury, angiogenesis and tumorigenesis [12], [13]. COX-2 is chronically overexpressed in many premalignant, malignant, and metastatic human cancers, and its expression is tightly controlled by the binding of multiple transactivators such as NF-κB. The transcription factor NF-κB includes two important members: p50 and p65. NF-κB regulates the transcription of a large number of genes, particularly those involved in immune, inflammatory and antiapoptotic responses [14]. The well-known anti-inflammatory substances such as glucocorticoids or nonsteroidal antiinflammatory drugs (NSAIDs) exert at least a portion of their effects by inhibiting NF-κB activity [15]–[17].

In this study, we isolated and identified effusanin E from *R. Serra* and analyzed its anticancer activity and elucidated the underlying mechanisms of its antitumor activities in NPC cells.

## Results

### Isolation and identification of effusanin E from *R. serra*

**Figure 1:**
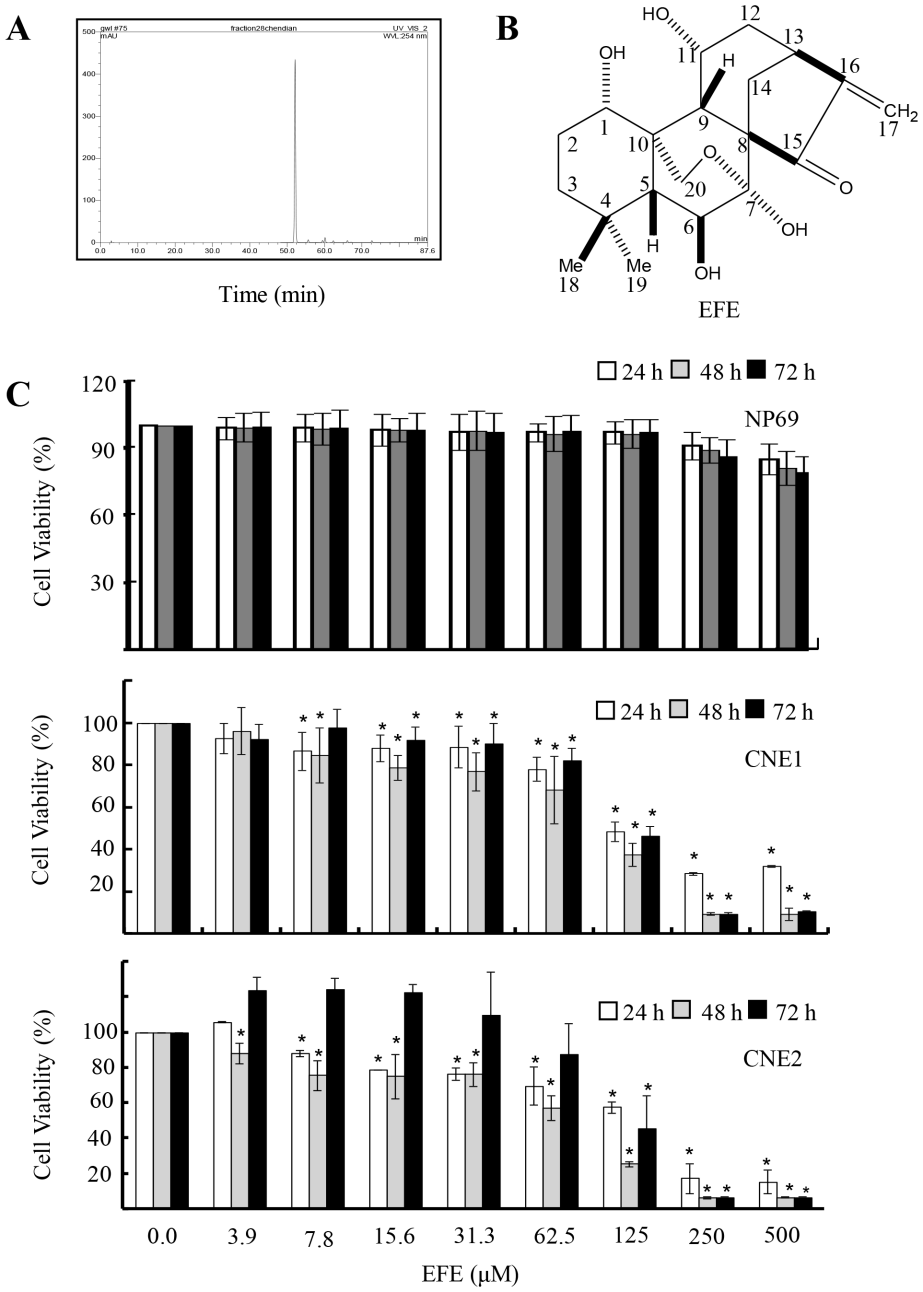
Purification and identification of effusanin E from *R. serra*. (**A**) HPLC analysis of effusanin E. (**B**) The structure of effusanin E (EFE). (**C**) Human CNE1 and CNE2 were treated with effusanin E (EFE) at 3.9–500 µM for 24 hours, 48 hours and 72 hours. The viability of the cells was determined using the MTS assay. Cells treated with the vehicle control (DMSO) were used as the reference group, with its cell viability set at 100%. The percent cell viability of each treatment group was calculated relative to that of cells treated with the vehicle control. The data are presented as the mean ± SD of three separate experiments. * represent P<0.05.

Effusanin E was purified and characterized according to our previous report [18] and obtained as a white, amorphous powder. HPLC analysis showed that the compound purified from *R. Serra* was pure (Fig. 1A). The mass spectrum (MS) and ^1^H and ^13^C NMR assays identified the chemical structure of the compound as effusanin E (Fig. 1B). Based on our data previously reported [18], the amount of effusanin E ranked second to rosmarinic acid and higher than other diterpenoids in *R. serra*, so effusanin E was one of the main substance from *R. Serra* [18]. The data of mass spectrum and ^1^H and ^13^C NMR of effusanin E were indicated below. Positive ESI MS m/z 387.1 [M+Na]^+^; ^1^H NMR (400 MHz, DMSO-d_6_): δ: 6.36 (1H, s, OH-7), 6.06 (1H, d, J = 10.3 Hz, OH-6), 5.78 (1H, s, H-17a), 5.50 (1H, s, H-17b), 5.34 (1H, d, J = 6.3 Hz, OH-11), 5.16 (1H, d, J = 4.5 Hz, OH-1), 4.33 (1H, m, H-11), 4.06 (1H, d, J = 10.4 Hz, H-20a), 3.87 (1H, dd, J = 1.74 Hz, J = 10.4 Hz, H-20b), 3.66 (1H, m, H-1), 3.58 (1H, dd, J = 5.9 Hz, J = 10.2 Hz, H-6), 3.10 (1H, dd, J = 4.1 Hz, J = 9.9 Hz, H-13), 2.60 (1H, m, H-12e), 2.12 (1H, d, J = 12.4 Hz, H-14e), 1.89 (1H, dd, J = 4.2 Hz, J = 12.4 Hz, H-14a), 1.54 (2H, m, H2-2), 1.43-1.38 (2H, m, H-3e and H-9), 1.24-1.19 (2H, m, H-3a and H-12a), 1.13 (1H, d, J = 5.6 Hz, H-5), 1.03 (3H, s, Me-18), 1.00 (3H, s, Me-19); ^13^C NMR (100 MHz, DMSO-d_6_): δ: 210.8 (C-15), 153.3 (C-16), 116.7 (C-17), 94.9 (C-7), 73.3 (C-6), 72.5 (C-1), 63.7 (C-20), 62.5 (C-11), 60.6 (C-5), 59.4 (C-8), 56.9 (C-9), 42.0 (C-10), 39.4 (C-3), 34.3 (C-13), 34.2 (C-4), 33.1 (C-19), 27.9 (C-2), 26.9 (C-14), 22.6 (C-18).

### Effusanin E inhibited cell proliferation

**Figure 2:**
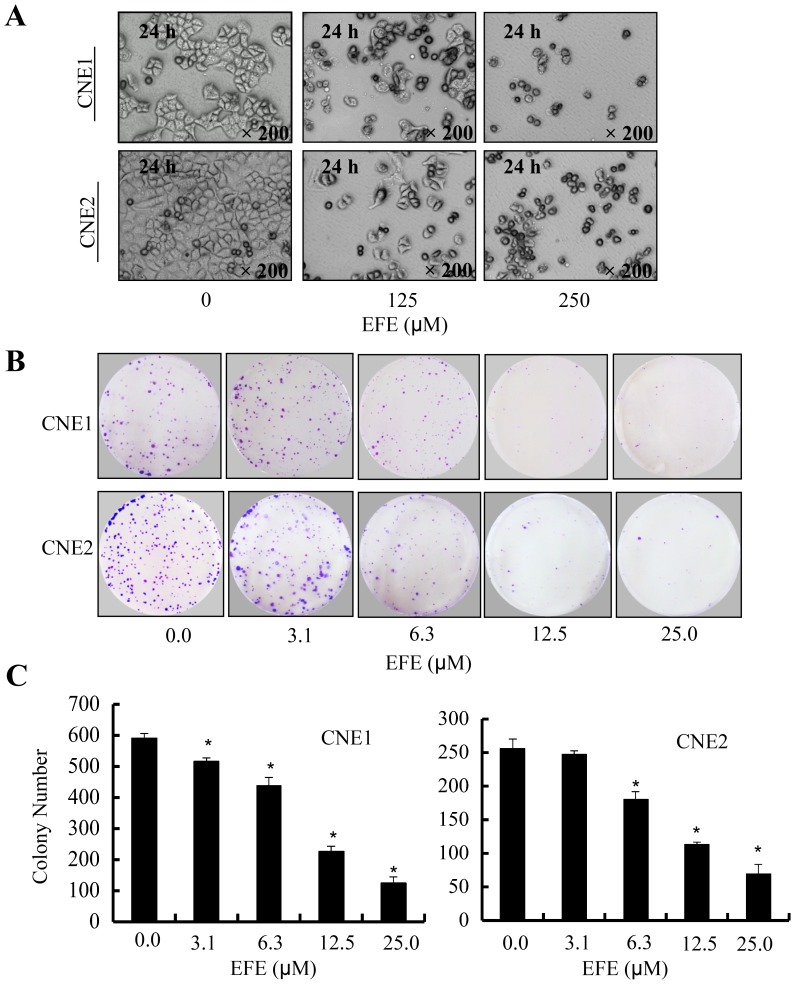
Effusanin E inhibits proliferation of NPC cells in vitro. (**A**) The cell morphology changes of human CNE1 and CNE2 cells after treatment with effusanin E (EFE) at 125 µM or 250 µM. (**B**) Colony formation assay of human CNE1 and CNE2 cells treated with effusanin E (EFE) at 3.1–25.0 µM. Cells were cultured as indicated in 6-well plates and treated with effusanin E for 10 days. After treament, the cells were dyed with crystal violet and took pictures. (**C**) The number of colony formation assay. The grown colonies of colony formation assay were scored. The data are presented as the mean ± SD of three separate experiments. * represent P<0.05.

The effect of effusanin E on the proliferation of NPC cells was investigated using MTS and colony formation assays. The result of the MTS assay showed that CNE1 and CNE2 cell viabilities were inhibited in a concentration-dependent manner in the presence of effusanin E while normal cell line NP69 cell viability was not affected (Fig. 1C). In addition, NPC cells became round, shrunken and detached from adjacent cells after 24 hours of treatment with effusanin E (Fig. 2A). To evaluate the long-term effects of effusanin E on NPC cell proliferation, colony formation was also carried out. The colony size (Fig. 2B) and number (Fig. 2C) were reduced by effusanin E in a dose-dependent manner. These results indicate that effusanin E inhibits cancer cell proliferation.

### Effusanin E induced apoptosis

**Figure 3:**
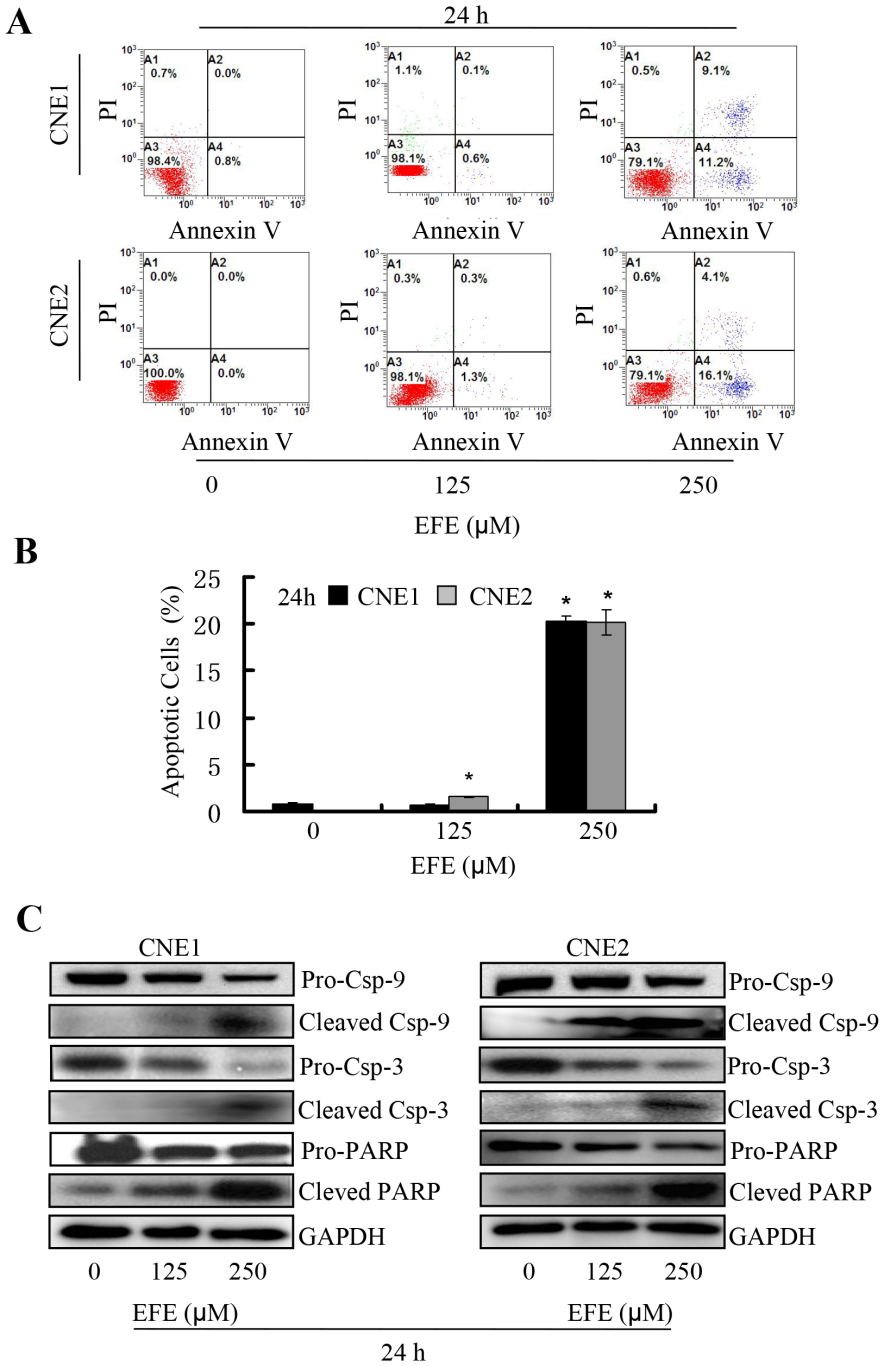
Effusanin E induced apoptosis. (**A**) Effusanin E (EFE) induced NPC cells apoptosis. Apoptosis was analyzed using TUNEL-based, fluorescence-activated cell sorter analysis and was represented by the relative percentages of TUNEL-positive cells versus those of DMSO-treated cells. (**B**) The percentages of apoptotic cells were calculated. (**C**) Western blotting of cleaved caspase-3, caspase-9 and PARP proteins in NPC cells. Total proteins isolated from the indicated cells were blotted with antibodies as labeled, and GAPDH was used as a control for sample loading.

To determine whether the cytotoxic effect of effusanin E was mediated via apoptosis, annexin V-FITC/PI double staining was performed. As shown in Fig. 3A and Fig. 3B, the percentage of apoptotic NPC cells increased from approximately 0.8% to 20.3% in CNE1, and from 0.0% to 20.2% in CNE2 after treatment with effusanin E from 0 µM to 250 µM.

We speculated that effusanin E affected the level of apoptotic genes. We therefore measured the expression of cleaved caspase-3/9 and cleaved PARP as well as pro- caspase-3/9 and PARP, and found that the expression of these apoptotic, cleaved proteins were increased significantly while the expression of pro-caspase-3/9 and PARP were decreased in NPC cells that were treated with effusanin E (Fig. 3C).

### Effusanin E inhibited COX-2 expression and promoter activity

**Figure 4:**
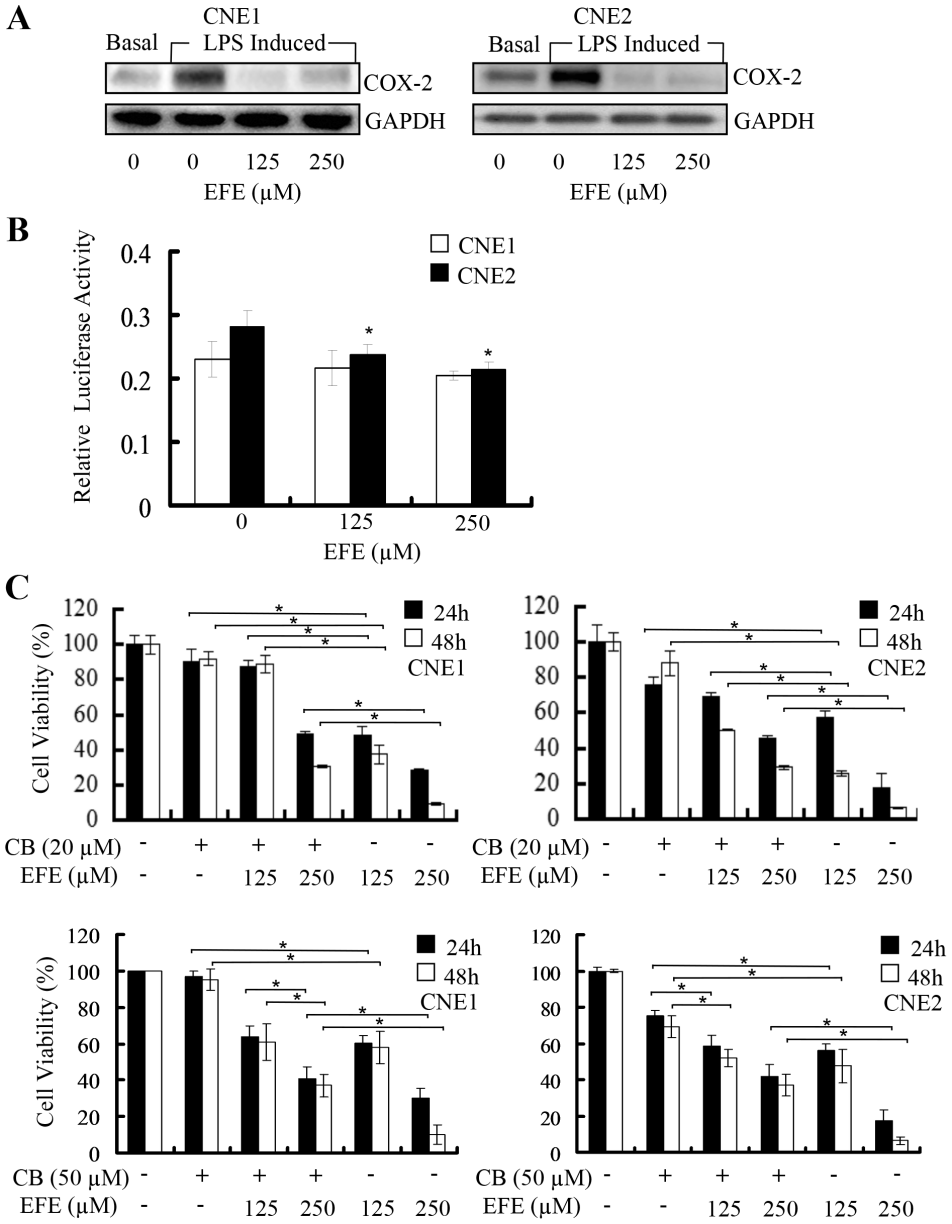
Effusanin E affects activity of the COX-2 pathway. (**A**) NPC cells were pretreated with lipopolysaccharides (LPS) (2 µg/ml) for 8 hours then treated with effusanin E at 125 µM and 250 µM (EFE 125 µM or EFE 250 µM). At 24 hours after treatment, COX-2 protein expression was determined by western blotting. (**B**) NPC cells were transfected with a luciferase expression vector containing a COX-2 5-flanking fragment for 8 hours and treated with effusanin E (EFE) at the indicated doses. At 24 hours after treatment, COX-2 promoter activities were determined. (**C**) The effect of effusanin E was inhibited by an inhibitor of COX-2 in NPC cells. NPC cells were treated with celecoxib (CB) (20 µM or 50 µM) for 8 hours followed by effusanin E at 125 µM and 250 µM (EFE 125 µM or EFE 250 µM) for up to 24 hours or 48 hours. At the indicated time points, the cells were analyzed using the MTS assay. The data are presented as the mean ± S.D. of three separate experiments. * represent P<0.05.

COX-2 is an inducible enzyme that plays a critical role in multiple pathophysiological processes such as inflammation, atherosclerosis, tissue injury, angiogenesis and tumorigenesis. Effusanin E has antibacterial, antiviral, anti-inflammatory and antitumor activities. Therefore, we verified that effusanin E inhibited COX-2 expression and found that effusanin E decreased the expression of COX-2 (Fig. 4A).

We first determined the effects of effusanin E on COX-2 promoter activity. We transfected NPC cells with a luciferase expression vector containing a 459-bp, COX-2 5-flanking fragment. The results indicated that the promoter activity was comparably inhibited by effusanin E in a dose-dependent manner in CNE2 cells (Fig. 4B).

To validate the role of effusanin E in regulating COX-2 signaling, we analyzed the effect of effusanin E on inhibition of cell proliferation by celecoxib, which is a COX-2-selective inhibitor. Pretreatment of the cells with this COX-2-selective inhibitor (20 µM or 50 µM) dramatically affected the effusanin E-mediated inhibition of proliferation at 24 hours and 48 hours after treatment (Fig. 4C).

### Effusanin E inhibited NF-κB signaling pathway

**Figure 5:**
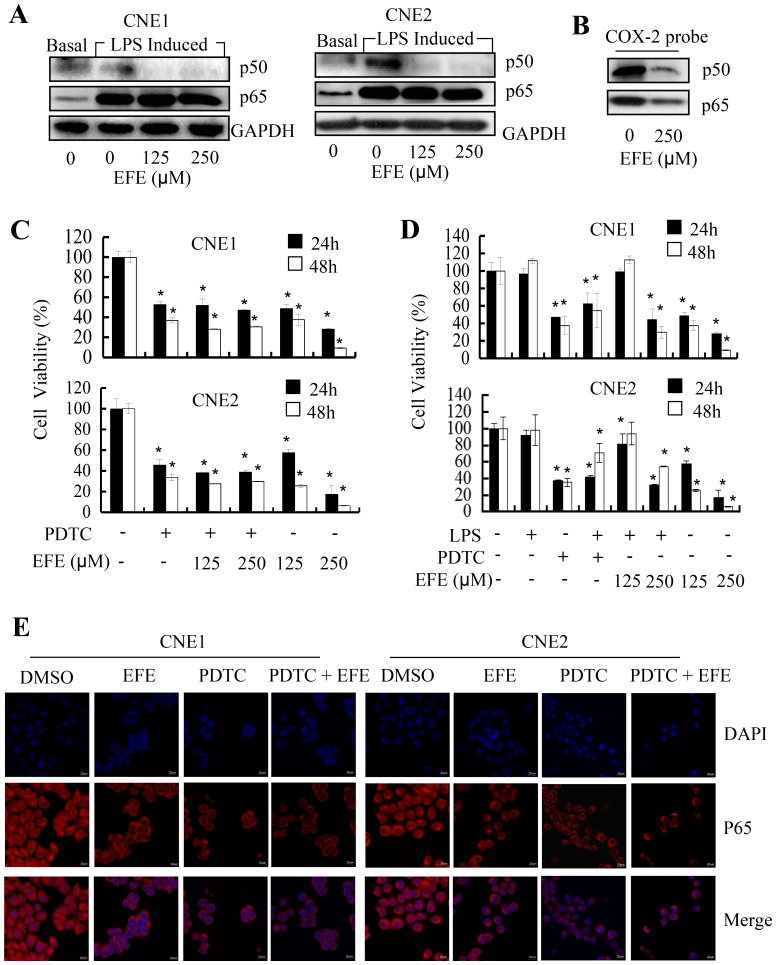
Effusanin E affects the activity of the NF-κB pathway. (**A**) Effusanin E inhibited the expression of p50 and p65. NPC cells were treated with DMSO (basal) or LPS (2 µg/ml) for 8 hours followed by effusanin E treatment at125 µM or 250 µM (EFE 125 µM or EFE 250 µM) for up to 24 hours. At the indicated time points, the cells were detected by Western blot analysis. (**B**) The binding of p50 and p65 NF-κB to the biotin-labeled, COX-2 promoter probe was analyzed by streptavidin-agarose pulldown assays, and the levels of p50 and p65 protein expression were detected by Western blot analysis. (**C**) The effect of effusanin E was inhibited by an inhibitor of NF-κB in NPC cells. NPC cells were treated with PDTC (100 µM) for 8 hours followed by effusanin E at 125 µM and 250 µM (EFE 125 µM or EFE 250 µM) treatment for up to 24 hours or 48 hours. At the indicated time points, the cells were analyzed using the MTS assay. (**D**) The effect of effusanin E was blocked by an activator of NF-κB. NPC cells were treated with LPS (2 µg/ml) or ammonium pyrrolidinedithiocarbamate (PDTC) (100 µM) for 8 hours followed by effusanin E at125 µM or 250 µM (EFE 125 µM or EFE 250 µM) treatment for up to 24 hours or 48 hours. At the indicated time points, the cells were analyzed using the MTS assay. (**E**) Analysis of the reduced nuclear translocation of NF-κB p65 by immunofluorescence imaging (IFI). CNE1 and CNE2 cells were treated with effusanin E at 125 µM (EFE) or PDTC (100 µM) or PDTC (100 µM) for 8 hours followed by effusanin E at 125 µM (PDTC + EFE), and NF-κB nuclear translocation in CNE1 and CNE2 cells was determined by immunofluorescence imaging analysis. * represent P<0.05.

Many studies have demonstrated that diverse inflammatory processes are regulated through NF-κB signaling. NF-κB pathways are known to promote proinflammatory cytokine gene expression, and previous studies showed that COX-2 expression is tightly controlled by the binding of multiple transactivators such as NF-κB. We further examined whether the inhibition of COX-2 signaling by effusanin E was mediated through NF-κB signaling in NPC cells. We determined the expression of p50/p65. As shown in Fig. 5A, effusanin E effectively blocked the expression of p50 but did not alter the expression of p65. We then pretreated NPC cells with PDTC (100 µM), a NF-κB-selective inhibitor, after effusanin E had been added at 125 µM and 250 µM for 8 hours. After 24 hours or 48 hours, the cell viabilities of the PDTC-alone and PDTC with effusanin E-treated cells were nearly the same. The results indicated that effusanin E significantly altered PDTC-mediated inhibition of NF-κB signaling (Fig. 5C).

NF-κB/COX-2 can be activated by LPS; therefore, we evaluated the effect of effusanin E on NF-κB/COX-2 activated by LPS. We pretreated NPC cells with LPS (2 µg/ml) for 8 hours and then with effusanin E at 125 µM and 250 µM. 24 hours or 48 hours later, the effect of effusanin E was strongly blocked at a concentration of 125 µM, which was consistent with the effect by LPS, and cell viabilities of cells treated with effusanin E and LPS at 250 µM were higher than those treated with effusanin E alone (250 µM) (Fig. 5D). These results showed that the effect of effusanin E toward NPC cells was greatly offset by LPS.

We then found reduced nuclear translocation of p65 in NPC cells by immunofluorescence imaging (Fig. 5E). Therefore, we determined whether the effusanin E-induced suppression of cell proliferation and COX-2 expression was mediated by inhibition of the binding of NF-κB to the COX-2 promoter in NPC cells. We analyzed the binding of p50 and p65 NF-κB to a biotin-labeled, 479-bp COX-2 promoter region that corresponded to its 5-flanking sequence by streptavidin-agarose pulldown assays, and we confirmed that the expression of p50/p65 was decreased (Fig. 5B). These results indicated that effusanin E suppressed COX-2 promoter activity by inhibiting p50/p65 binding to the COX-2 promoter, thus leading to decreased cell survival, and proliferation of NPC cells. These results demonstrate the antitumor effects that are exerted by effusanin E through the modulation of the NF-κB/COX-2 signaling pathways in Human Nasopharyngeal Carcinomas.

### Effusanin E inhibited tumor growth in a xenograft mouse model

**Figure 6:**
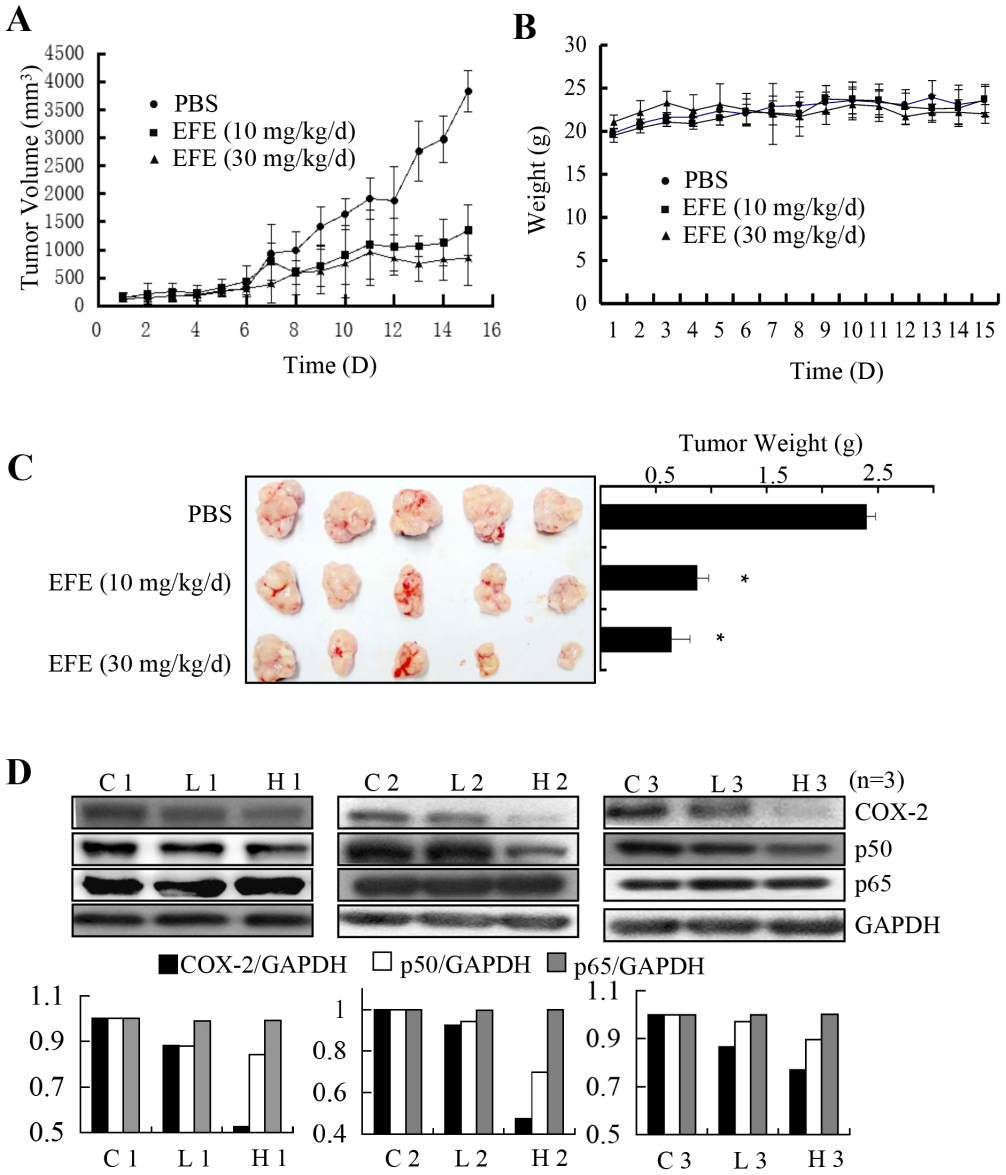
Effusanin E suppressed tumor growth in a human NPC nude mice model. (**A**) Tumor volumes measured *in situ* and images of the mice that were treated with the negative control (20 µl DMSO and 80 µl PBS) or effusanin E (EFE) at the indicated doses. The mice were randomly separated into three groups and treated with the negative control or effusanin E, and the tumor volumes were measured every other day. (**B**) Weight changes of the nude mice were measured after treatment every other day. (**C**) The weight of the tumors from the mice and images of tumors that were removed from the mice. After the mice were sacrificed, the tumors were removed from the mice and pictures were taken. (**D**) Representative Western blot showing the expression of COX-2, p50 and p65 in the tumors from nude mice. C1, C2 and C3 represent tumor 1, tumor 2 and tumor 3 from negative control. L1, L2 and L3 represent tumor 1, tumor 2 and tumor 3 from effusanin E-treated mice at 10 mg/kg/d. H1, H2 and H3 represent tumor 1, tumor 2 and tumor 3 from effusanin E-treated mice at 30 mg/kg/d. * represent P<0.05.

To directly evaluate the effect of effusanin E on tumors in vivo, tumor growth of CNE2 cells in nude mice were tested using a tumor xenograft model. When the tumors became palpable (∼200 mm^3^), the mice were treated with either a vehicle control or effusanin E at 10.0 mg/kg/d or 30.0 mg/kg/d for 30 days. The results showed that tumor growth was decreased and slower after effusanin E treatment when compared with the vehicle control. During the analysis, no significant weight changes in the mice were observed (Fig. 6B). These results suggest that effusanin E did not induce significant weight loss and had no obvious toxicity. Greater and heavier in vivo tumor formation and volume were observed in the control mice compared to mice treated with effusanin E (Fig. 6A). After the mice were sacrificed, the tumors were removed from the mice and pictures were taken. The weight of the tumors was showed in Fig. 6C. Thus, effusanin E was able to inhibit the growth of NPC tumor in vivo. Furthermore, the expression of COX-2 and p50 were downregulated in the tumor, while the expression of p65 did not alter (Fig. 6D), these results are consistent with cell study.

## Discussion

In this study, we demonstrate that effusanin E inhibited the proliferation and induced apoptosis of NPC cells in vitro. Our study showed that effusanin E inhibited NF-κB signaling, furthermore, effusanin E reduced the tumor volume and weight of treament mice compared with the control group. In detail, effusanin E inhibited the p50 protein level and reduced nuclear translocation of p65. Pretreatment with a NF-κB-selective inhibitor had an additive effect on the effusanin E-mediated inhibition of proliferation and the nuclear translocation of p65, while pretreatment with LPS abrogated the effusanin E-mediated inhibition of proliferation. We also found that effusanin E suppressed COX-2 promoter activity and inhibited p50/p65 binding to the COX-2 promoter, thus leading to decreased cell proliferation, and induced apoptosis. Also effusanin E stimulated PARP, caspase-3 and -9 cleavages. To our knowledge, this is the first report that demonstrated effusanin E-exerted antitumor effects through the modulation of NF-κB signaling pathways and induced apotosis in NPC cells.

The use of celecoxib is associated with a significant decrease in many cancer risk, including nasopharyngeal carcinoma. However, the long-term use of high-dose celecoxib might be limited owing to cardiovascular side effects [19]. In this study, we found that effusanin E, extract from a Chinese herb, could reduce celecoxib dose and potentiate the growth-inhibitory effect in NPC cells.

The expression of COX-2 plays a key role in inflammatory disorders and cancers [20]. COX-2 expression is strongly correlated with prognosis in some cancers [21]. COX-2 inhibition may result in cell-growth suppression and apoptosis. A core promoter region that lies within 500 bp from the COX-2 transcription start site harbors several regulatory elements, notably the cyclic adenosine monophosphate response element, CCAAT/enhancer binding protein enhancer element and NF-κB binding sites, which are essential for COX-2 promoter activity in response to inflammatory signals [22]. The most abundant NF-κB dimer is the p65/p50 heterodimer followed by the p50/p50 and the p65/p65 homodimer complexes [23], [24]. Aberrant regulation of NF-κB affects all six hallmarks of cancer through the regulation of genes that are associated with cell proliferation, angiogenesis, metastasis, tumor promotion, inflammation and the suppression of apoptosis [25]. Currently, a large number of natural compounds have been reported as NF-κB inhibitors [26], [27]. It is known that oridonin, ponicidin (two 7,20-epoxy-*ent*-kaurenoids), xindongnin A and xindongnin B (two C-20-nonoxygenated-*ent*-kauranoids) could be potential inhibitors of NF-κB transcription activity by inhibiting the NF-κB DNA-binding activity and the expression of its downstream target COX-2 [28], this finding was consistent with our results . It is well known that p65 is a critical transactivation subunit of NF-κB [29], [30], and our study showed that effusanin E apparently blocked with translocation of p65, which is supported by the report that oridonin, ponicidin, and kamebakaurin have an impact on the translocation of p65 to different degrees. It also had been reported that diterpenoids (oridonin) derived from I. Japonicus exert its anti-cancer properties by regulating NF-κB signaling pathways [8]–[10], which support our result suggesting that diterpenoids are a new class of NF-κB inhibitor. Moreover, it is known that effusanin C inhibits inflammatory responses via blocking NF-κB signaling in monocytes [11], which confirms that effusanin E could inhibit tumor cells by targeting NF-κB. To confirm the COX-2/NF-κB signaling pathway was involved in the effusanin E-mediated inhibition of cell viability, we pretreated the cells with COX-2 or NF-κB inhibitor (CB or PDTC) for 8 h, and then treated with effusanin E for 24 h. The results showed that the combined treatment of CB or PDTC with effusanin E did not significantly increase the inhibition of cell viability as compared with the effusanin E alone. This maybe because pretreatment with CB or PDTC effectively abrogated or completely blocked the COX-2 and NF-κB signaling, and while effusanin E (125 µM or 250 µM) was added after CB or PDTC treatment, effusanin E at this concentration could not function to inhibit cell viability by suppressing the COX-2 and NF-κB signaling. These results suggested that effusanin E inhibits tumor cell growth partially through the inhibition of the NF-κB/COX-2 signaling pathway in NPC cell signaling pathways. *R. serra* is famous for its antibacterial, antiviral, anti-inflammatory and antitumor activities, and effusanin E, a compound from natural herb, inhibited NPC cells via disrupting NF-κB signaling and induced apotosis in NPC cells, meanwhile, effusanin E significantly suppressed tumor growth in a xenograft mouse model of NPC cells without obvious toxicity, moreover, the expression of p50 and COX-2 were downregulated in the tumors of nude mice, which are consistent with cell study, so these findings evidenced the antitumor activities of *R. serra*. However, the upstream of NF-κB remains unknown, and needs further study to research. We also selected the 500-bp COX-2 promoter probe which included two conserved NF-κB-binding sites and found that effusanin E decreased the binding of NF-κB on the COX-2 promoter. We will use ChIP assays to confirm this in our further study.

In summary, this report is to study the anticancer mechanism of effusanin E from *R. serra*, and the results showed that effusanin E suppresses nasopharyngeal carcinoma growth by inhibiting NF-κB signaling. Therefore, effusanin E is a promising natural compound for assisting NPC treatment. Furthermore, this study confirms that Xihuangcao, an herbal tea, is capable of anticancer activity.

## Materials and Methods

### Ethics Statement

The study was approved by the Ethics Committee of Sun Yatsen University Cancer Center. The aerial parts of R. serra were collected from Luofu mountain (GPS coordinates: 23.29522, 114.105266), Huizhou, Guangdong, China on September 14th, 2011, and were authenticated by Professor Huagu Ye of South China Botanical Garden, Chinese Academy of Sciences, where voucher specimens (voucher specimen number 21373) were kept. R. serra leaf was separated from stem, cleanly washed without any damage, and sun-dried and ground into fine powder by laboratory mill (FW100, Taisite Instrument Co., Ltd, Tianjin, China). And this study was carried out in strict accordance with the recommendations in the Guide for the Care and Use of Laboratory Animals of the National Institutes of Health. The protocol was approved by the Committee on the Ethics of Animal Experiments of Sun Yat-Sen University (Permit Number: SCXK (yue) 2008–0002). All efforts were made to minimize suffering. The nude mice were employed for tumorigenicity analysis. No specific permissions were required for these locations/activities. In addition, the field studies did not involve endangered or protected species.

### Cell lines and cell culture

The human nasopharyngeal high differentiated squamous epithelium carcinoma cell (CNE1), human nasopharyngeal low differentiated squamous epithelium carcinoma cell (CNE2) lines and normal nasopharyngeal epithelial cell line NP69 were provided by Sun Yat-Sen University Cancer Center (China) and were cultured in DMEM medium (Invitrogen, US) that was supplemented with 10% fetal bovine serum (FBS) (HyClone, Logan, UT) and were maintained in an incubator with a humidified atmosphere of 95% air and 5% CO_2_ at 37°C.

### Reagents

Ammonium pyrrolidinedithiocarbamate (PDTC), celecoxib (CB) and lipopolysaccharides (LPS) were purchased from Sigma (St. Louis, MO). Effusanin E was purified from *R. serra* by column chromatography in our previous study. The purity of the compound exceeds 95%.

### Plant material

The aerial portions of *R. serra* were collected from Luofu mountain (GPS coordinates: 23.29522, 114.105266), Huizhou, Guangdong, China on September 14^th^, 2011, and were authenticated by Professor Huagu Ye of South China Botanical Garden, Chinese Academy of Sciences, where voucher specimens (voucher specimen number 21373) were kept. *R. serra* leaf was separated from stem, cleanly washed without any damage, and sun-dried and ground into fine powder by laboratory mill (FW100, Taisite Instrument Co., Ltd, Tianjin, China). No specific permissions were required for these locations or activities. In addition, the field studies did not involve endangered or protected species.

### Cell viability assay

The cell viability was determined using the MTS assay. Cells were plated in 96-well plates (2000 cells/well) and were treated with the tested samples at the indicated doses. At 24, 48 or 72 hours after treatment, 10 µl of MTS was added into each well, and the cell viability was determined at 490 nm.

### Cellular morphology observation

Cells were treated with effusanin E at the indicated doses. After 24 hours, cellular morphology was observed using an Olympus microscope that was fitted with a digital camera.

### Colony formation assay

Cells were seeded into a 6-well culture dish (800 cells/well). After incubation for 24 hours, effusanin E was added to the cells. Once colony formation was observed visually, the cells were washed twice with PBS, fixed for 30 min at room temperature with 4% paraformaldehyde and stained with Crystal Violet Staining Solution for 10 min. Subsequently, the cells were washed an additional two times with PBS. Colonies of >50 cells were counted and analyzed using a microscope, and the plate colony formation efficiency was calculated using the following formula: plate colony formation efficiency  =  (number of colonies/number of cells inoculated) ×100%.

### Apoptosis assay

The effusanin E-treated cells (2×10^4^) were stained with AnnexinV-FITC using an Annexin V/Dead Cell Apoptosis Kit (Invitrogen, USA). The cells were washed twice in PBS and re-suspended in binding buffer. The suspension (100 µl) was then stained with 5 µl of Annexin V-FITC and gently vortexed and incubated in the dark for 15 minutes at room temperature. Last, 10 µl of Propidium Iodide (PI) was added, and the suspension was incubated in the dark for 5 minutes at room temperature. After the addition of 400 µl of binding buffer to each tube, the cells were analyzed by flow cytometry (Cytomics FC500 Flow Cytometry; Beckman Coulter).

### Western blot analysis

Cells were treated with effusanin E at the indicated doses for 24 hours, and cell lysates were subjected to Western blot analysis using antibodies for GAPDH, p50 and p65 NF-κB (Santa Cruz Biotechnology, Santa Cruz, CA) and antibodies for caspase-3, caspase-9, PARP, cleaved caspase-3, cleaved caspase-9, cleaved PARP and COX-2 from Cell Signaling Technology (Beverly, MA).

The tumor of nude mice were dissected and homogenized, then the sample of protein were subjected to Western blot analysis using antibodies for GAPDH, p50 and p65 NF-κB from Santa Cruz Biotechnology (Santa Cruz, USA) and COX-2 from Cell Signaling Technology (Beverly, MA, USA).

### Validation of the regulation of COX-2 and NF-κB signaling by effusanin E

To validate the role of effusanin E in regulating NF-κB signaling, the effect of effusanin E on the PDTC- (a NF-κB-selective inhibitor), celecoxib- (a COX-2-selective inhibitor) or LPS-mediated inhibition of cell proliferation was analyzed. NPC cells plated in 96-well plates (2000 cells/well) were pretreated with PDTC (100 µM), celecoxib (20 µM or 50 µM) or LPS (2 µg/mL) for 8 hours and then treated with effusanin E at 125 µM or 250 µM. After treatment for 24 hours, cell viability was determined using MTS analysis.

### Analysis of reduced nuclear translocation of p65 by immunofluorescence imaging (IFI)

Cells were seeded in six-well chamber slides at a density of 1×10^5^ cells per well and treated with effusanin E (125 µM) or PDTC or PDTC followed by effusanin E at 125 µM. After 24 hours, the cells were washed in PBS, fixed at room temperature with 4% paraformaldehyde, and permeabilized with 0.2% Triton X-100. Immunofluorescence staining was performed according to standard procedures. Briefly, the treated cells were first stained with the anti-p65 antibody followed by incubation with FITC-conjugated anti-rabbit IgG secondary antibody and co-staining with DAPI.

### Analysis of promoter activity

A promoter region of the human COX-2 gene (459 bp) and its fragment deletions were cloned into the luciferase reporter vector pGL3. COX-2 promoter activity was determined by transient expression of the luciferase vectors in cells using Lipofectamine 2000 (Invitrogen). Luciferase activity was measured using an assay kit (Promega, Madison, WI, USA).

### DNA–protein binding by streptavidin-agarose pulldown assay

Transactivator binding to COX-2 core promoter probes were determined using a streptavidin-agarose pulldown assay as previously described [31]. A 479-bp, biotin-labeled, double-stranded probe corresponding to the COX-2 promoter sequence (−30 to −508) was used for the experiments, and DNA-bound NF-κB was analyzed by Western blotting.

### Animal assays

This study was carried out in strict accordance with the recommendations in the Guide for the Care and Use of Laboratory Animals of the National Institutes of Health. The protocol was approved by the Committee on the Ethics of Animal Experiments of Sun Yat-Sen University (Permit Number: SCXK (yue) 2008-0002). All efforts were made to minimize suffering. The nude mice were employed for tumorigenicity analysis. Male, nude mice, 4-5 weeks old, weighting 17–22 g were housed in filter-capped cages kept in a sterile facility and maintained in a specific, pathogen-free barrier system. CNE2 cells were harvested by 0.25% trypsinization, washed twice with sterile PBS and resuspended at a concentration of 1×10^7^ cells/ml. CNE2 cells (100 µl) were injected subcutaneously into the flank of nude mice, and the mice were randomly assigned to receive either 20 µl DMSO and 80 µl PBS (control; 5 mice) or effusanin E (10 mg/kg/d or 30 mg/kg/d, 20 µl effusanin E solution and 80 µl PBS; 10 mice). Treatment was administered through intraperitoneal injection, and tumor growth was measured after the injection and then every day. The tumor volume (V) was monitored by measuring its length (L) and width (W) with calipers and calculated using the following formula: V = (L×W^2^)×0.5. Photographs were taken 16 days after the injection. Finally, animals were euthanized by CO_2_ asphyxiation.

### Statistical analysis

The significance was evaluated by the paired *t* test, and * represent P<0.05, they were considered to be a statistically significant difference between the treatment and control groups. SPSS 11.0 software was used for all statistical analysis. All the experiments were performed three times, and the mean values and standard deviations were calculated.
